# Comparing complaint-based triage scales and early warning scores for emergency department triage

**DOI:** 10.1136/emermed-2021-211544

**Published:** 2022-04-13

**Authors:** Michiel Schinkel, Lyfke Bergsma, Lars Ingmar Veldhuis, Milan L Ridderikhof, Frits Holleman

**Affiliations:** 1 Center for Experimental and Molecular Medicine, Amsterdam UMC location University of Amsterdam, Amsterdam, Netherlands; 2 Internal Medicine, Amsterdam UMC, Location VUmc, Amsterdam, The Netherlands; 3 Emergency Medicine, Amsterdam UMC, Location AMC, Amsterdam, The Netherlands

**Keywords:** triage, emergency department

## Abstract

**Background:**

Emergency triage systems are used globally to prioritise care based on patients’ needs. These systems are commonly based on patient complaints, while the need for timely interventions on regular hospital wards is usually assessed with early warning scores (EWS). We aim to directly compare the ability of currently used triage scales and EWS scores to recognise patients in need of urgent care in the ED.

**Methods:**

We performed a retrospective, single-centre study on all patients who presented to the ED of a Dutch Level 1 trauma centre, between 1 September 2018 and 24 June 2020 and for whom a Netherlands Triage System (NTS) score as well as a Modified Early Warning Score (MEWS) was recorded. The performance of these scores was assessed using surrogate markers for true urgency and presented using bar charts, cross tables and a paired area under the curve (AUC).

**Results:**

We identified 12 317 unique patient visits where NTS and MEWS scores were documented during triage. A paired comparison of the AUC of these scores showed that the MEWS score had a significantly better AUC than the NTS for predicting the need for hospital admission (0.65 vs 0.60; p<0.001) or 30-day all-cause mortality (0.70 vs 0.60; p<0.001). Furthermore, when non-urgent MEWS scores co-occur with urgent NTS scores, the MEWS score seems to more accurately capture the urgency level that is warranted.

**Conclusions:**

The results of this study suggest that EWSs could potentially be used to replace the current emergency triage systems.

**Key messages**

**What is already known on this topic**

- Complaint-based triage scales are the norm in ED triage. However, their performance has shown to be highly variable and their practicality has been questioned due to their complexity.
- Early warning scores have been shown to have good predictive value for admission and hospital outcome.

**What this study adds**

- In this retrospective, single-centre study comparing a complaint-based triage scale with an early warning score, we found that an early warning score was a better discriminator for admission and 30-day mortality than the Netherlands Triage Score.
- In cases where these approaches yield strikingly different urgency scores, the early warning score was a better predictor.

**How this study might affect research, practice or policy**

- This study suggests that early warning scores could potentially replace current emergency triage systems.

## Introduction

Over the past decades, ED presentation rates have increased worldwide.^1^ At times of supply and demand mismatches, medical resources should be allocated based on the patients’ needs to ensure patient safety.^1 2^ Emergency triage systems are used globally to assess these specific needs.

The performance of any emergency triage system is dependent on the environment in which it is used. Therefore, most countries use modified international triage systems to fit their particular situation. Commonly known triage scales include the internationally used Emergency Severity Index (ESI), the UK-based Manchester Triage Scale (MTS) and the Canadian Triage and Acuity Scales (CTAS).^3^ In Holland, the Netherlands Triage System (NTS) is used, which is a modified version of the MTS.^4^ A common theme among all triage systems is that these are decision trees based on patient complaints. Specific symptoms or high pain scores will result in higher urgency levels. Recently, two large systematic reviews have shown that the performance of triage scores varies considerably and that a significant part of the population may not be designated to the appropriate acuity group.^5 6^ Furthermore, there has been debate over the impractical complexity of the current triage systems and the need to rethink ED triage.^7^

The complaint-based approach during emergency triage is noticeably different from the simple early warning scores (EWS) used to detect clinical deterioration and the need for timely intervention in patients admitted to in-hospital wards. In the Netherlands, the Modified Early Warning Score (MEWS) is used in this regard.^8^ The EWS scores can accurately detect patients at high risk of deterioration and have been studied in numerous settings.^9–14^ Although EWS scores have been extensively studied for use in ED triage, they were never specifically developed to be triage tools.^15–22^ Furthermore, EWS scores and triage scales have not been compared head-to-head.

In this study, we aim to compare the ability of currently used triage scales and EWS scores to recognise patients in need of urgent care in the ED. These two approaches will be represented by the NTS and MEWS scores, respectively, as they are commonly used in the Netherlands.

## Methods and study design

### Study setting

A retrospective, single-centre study was performed using data from the electronic health records (EHRs) of the Amsterdam UMC, location Vrije Universiteit Medical Center (VUmc). Data recorded between 1 September 2018 and 24 June 2020 were extracted. Data from before September 2018 could not be used since the storage of the NTS form was outsourced until this point in time. The VUmc is a Level 1 trauma centre and teaching hospital with an estimated 29 000 ED presentations annually. The study adheres to the ‘Standards for Reporting Diagnostic Accuracy’ (STARD) guideline.^23^

### Patient selection

We included all patients who presented to the ED of Amsterdam UMC, location VUmc, and for whom an NTS score as well as a MEWS score was documented. Patients under the age of 18 were excluded, as were patients with an NTS score of 0. The NTS score of 0 indicates that the patient was being resuscitated on arrival, which makes triage redundant.

### NTS and MEWS measurements

All patients in the VUmc are triaged by a triage nurse who documents an NTS score. The NTS is a standardised five-level protocol with questions regarding patient complaints and pain levels. Lower numbered urgency levels (eg, NTS 1 or 2) indicate higher urgency^4^ (online supplemental table 1).

10.1136/emermed-2021-211544.supp1 Supplementary data

The MEWS score is also frequently documented as part of the initial work-up in our hospital’s ED, but is not used to decide on the urgency level and is therefore not mandatory. The MEWS is derived from seven parameters (systolic BP, HR, RR, temperature, peripheral oxygen saturation, level of consciousness and urine production).^24^ Also, an additional point may be scored when the nurse is particularly worried (online supplemental table 2). The higher the MEWS scores, the more likely a patient is to deteriorate. Prior studies report that MEWS scores of 5 or higher are critical and indicate a high likelihood of deterioration, while Dutch hospitals are prompted to use a cut-off of 3.^8 24 25^

### Outcome measures

Surrogate outcomes for high urgency were used, as is frequently done with the development and assessment of triage tools, since no gold standard for urgency exist.^26^ The outcomes we studied were admission rates and 30-day all-cause mortality, since they were clearly defined in the EHR data and are among the most studied surrogates in this regard.^4 26^

### Statistical analysis

The characteristics of the study population are presented with descriptive statistics. Categorical variables are presented as counts and percentages. Normality of the data is assessed using histograms and Q-Q plots. Non-normally distributed continuous data are presented with medians and IQRs. NTS and MEWS scores are presented using bar charts and cross-tables. To assess for selection bias, we determined the distribution of NTS scores in the population studied as well as the entire adult population seen in the ED during the study period

The predictive performance of both scores for the primary outcomes are visualised using receiver operating characteristics (ROC) curves and corresponding areas under the curve (AUCs). To compare the NTS and MEWS scores, we use the DeLong’s test for the comparison of AUCs of two correlated ROC curves.

Data analysis was performed using R V.3.6.3 (R Foundation of Statistical Computing, Vienna, Austria).^27^ The figures were created using the ‘ggplot2’ package,^28^ and the paired AUC analysis was done using the ‘pROC’ package.^29^

### Patient and public involvement

No patient involved.

## Results

### Baseline characteristics

We identified 55 086 ED visits by 39 907 unique adult patients between 1 September 2018 and 24 June 2020. In 53 106 of these visits, the NTS triage score was recorded. Of these patients, 12 452 patients had a documented MEWS score. After exclusion of patients with an NTS score of 0, the final study population consisted of 12 317 unique visits. Table 1 shows the baseline characteristics of this study population.

**Table 1:** Baseline characteristics of the study population

Characteristic	Total (n=12 317)
Age (years, median (IQR))	60 (41–73)
Female, n (%)	5847 (47.5)
Chief complaint*, n (%)	
Stomach aches	1313 (10.7)
General malaise	1143 (9.3)
Shortness of breath	757 (6.1)
Chest pain	670 (5.4)
Neurological deficit	601 (4.9)
ED length of stay (hours, median (IQR))	3.8 (2.6–5.3)
Admitted, n (%)	5004 (40.6)
30-day mortality, n (%)	476 (3.9)

*Only complaints with a frequency over 500 are presented.

### Frequency distributions of NTS and MEWS scores

**Figure 1:**
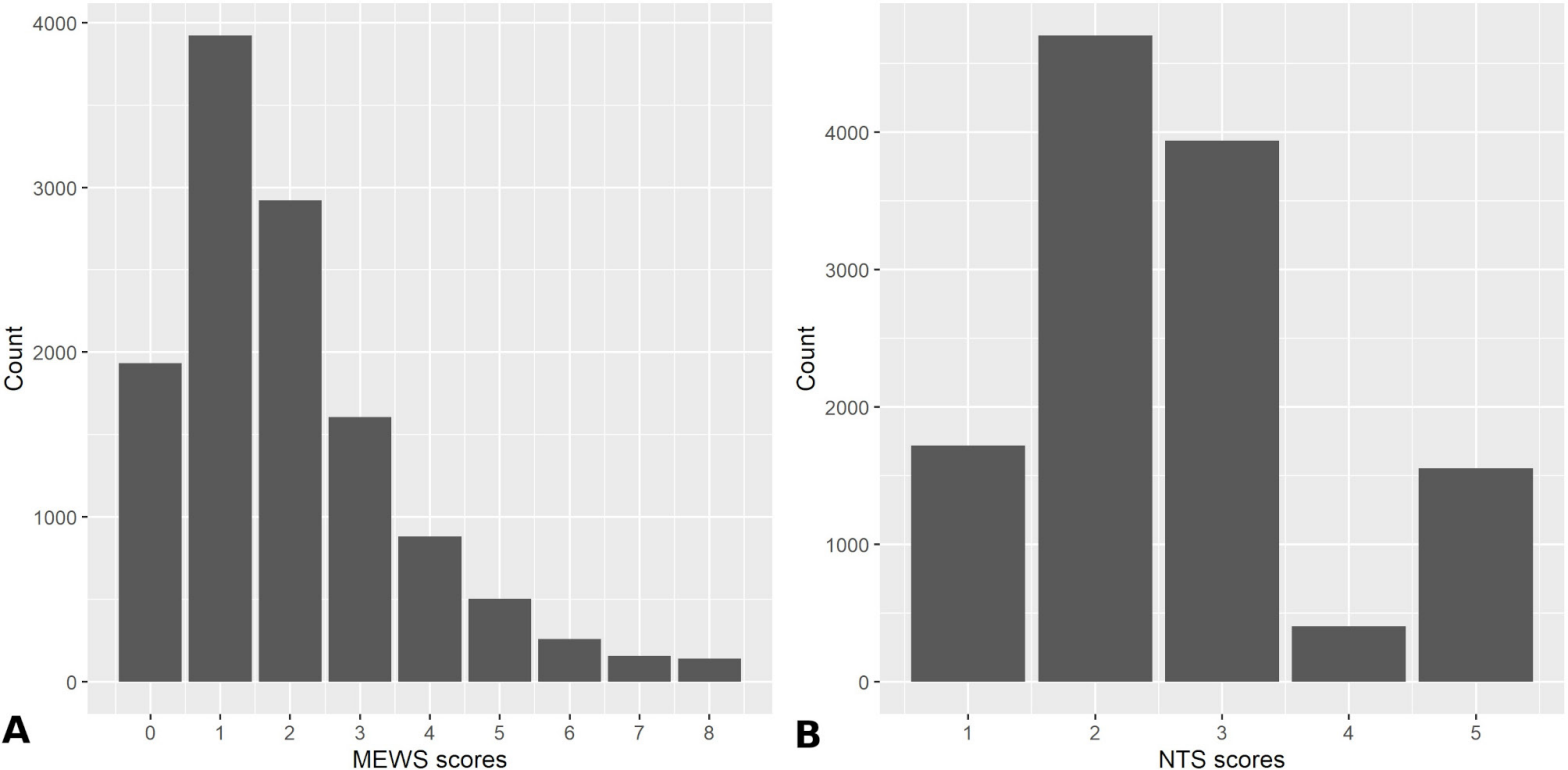
A bar chart of the absolute counts of the various Netherlands Triage System (NTS) scores (A) and Modified Early Warning Scores (MEWS) (B) in the study population.

In figure 1A, we present the absolute counts of the various NTS scores. Notably, the NTS scores do not seem to follow any particular distribution; the majority of patients are assigned levels 2 and 3, and the NTS score of 4 is infrequently given. Similar results were seen in the complete population before excluding any patient (online supplemental figure 1). The MEWS scores follow a clear right-skewed distribution (figure 1B).

### Comparison of NTS and MEWS scores

**Figure 2:**
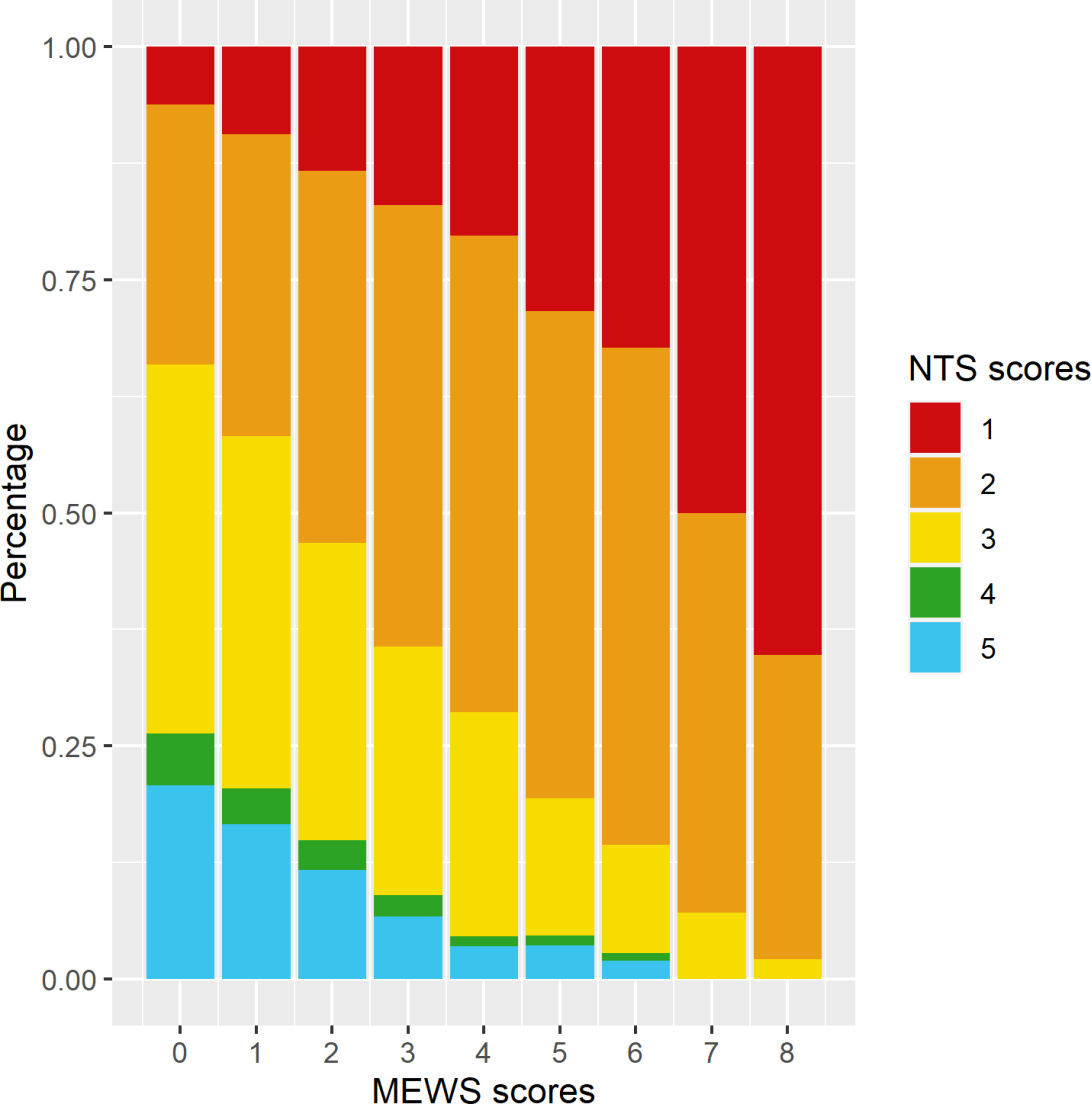
Modified Early Warning Scores (MEWS) and Netherlands Triage System (NTS) scores compared.

Generally, the proportion of lower (more urgent) NTS scores increases with increasing (more urgent) MEWS scores (figure 2). In table 2, we present the counts of the different combinations of NTS and MEWS scores assigned. Notably, high NTS scores (non-urgent) never co-occur with high (urgent) MEWS scores, while low (more urgent) NTS scores do co-occur with low (non-urgent) MEWS scores. For example, the combination of NTS 1/MEWS 0 is reported in 120/12 317 (1%) instances and the combination of NTS 1/MEWS 2 in 388/12.317 (3.2%).

**Table 2:** Frequencies of patients with all different Modified Early Warning Score (MEWS) and Netherlands Triage System (NTS) combination

MEWS	NTS					
	1	2	3	4	5	Total
0	120	539	765	108	401	1933
1	366	1271	1482	151	651	3921
2	388	1168	932	92	341	2921
3	272	761	428	37	107	1605
4	179	451	212	9	31	882
5	142	262	74	5	18	501
6	83	137	30	2	5	257
7	78	67	11	0	0	156
8 or above	92	46	3	0	0	141
**Total**	1720	4702	3937	404	1554	12 317

In tables 3 and 4, we demonstrate the outcomes of patients with each combination of NTS and MEWS scores. Where the NTS was notably more urgent than the MEWS, the admission and mortality rates are lower than the average in the population. In the above example of an NTS 1 of 1 and MEWS of 0, the admission rate (34%) and mortality rate (2%) are lower than the average admission rate of 40.6% and mortality rate of 3.9%.

**Table 3:** Fraction of patients admitted to the hospital stratified based on their Modified Early Warning Score (MEWS) and Netherlands Triage System (NTS) score

MEWS	NTS					
	1	2	3	4	5	Average
0	0.34	0.29	0.25	0.21	0.19	0.25
1	0.35	0.34	0.34	0.23	0.19	0.31
2	0.46	0.45	0.41	0.30	0.23	0.41
3	0.52	0.53	0.50	0.35	0.30	0.50
4	0.63	0.64	0.56	0.56	0.20	0.60
5	0.73	0.66	0.65	0.80	0.44	0.67
6	0.80	0.78	0.57	1.00	0.60	0.76
7	0.82	0.78	0.64	NA	NA	0.79
8 or above	0.78	0.83	1.00	NA	NA	0.80
**Average**	0.53	0.46	0.38	0.27	0.21	0.41

NA, not available.

**Table 4:** Fraction of patients who died within 30 days stratified based on their Modified Early Warning Score (MEWS) and Netherlands Triage System (NTS) score

MEWS	NTS					
	1	2	3	4	5	Average
0	0.02	0.01	0.02	0.01	0.01	0.01
1	0.02	0.03	0.02	0.03	0.01	0.02
2	0.02	0.03	0.04	0.04	0.03	0.03
3	0.03	0.05	0.05	0.03	0.04	0.05
4	0.10	0.06	0.05	0.11	0.06	0.07
5	0.11	0.09	0.07	0.20	0.06	0.09
6	0.23	0.07	0.07	0.00	0.20	0.12
7	0.18	0.16	0.09	NA	NA	0.17
8 or above	0.23	0.28	0.34	NA	NA	0.25
**Average**	0.07	0.04	0.03	0.03	0.02	0.04

NA, not available.

Further, for any NTS score, the admission and mortality ranges can vary greatly for different MEWS scores in those same patients. For example, for patients with an NTS score of 2 the admission rate ranged from 29% to 83% depending on the MEWS score.

### Paired AUC analysis

**Figure 3:**
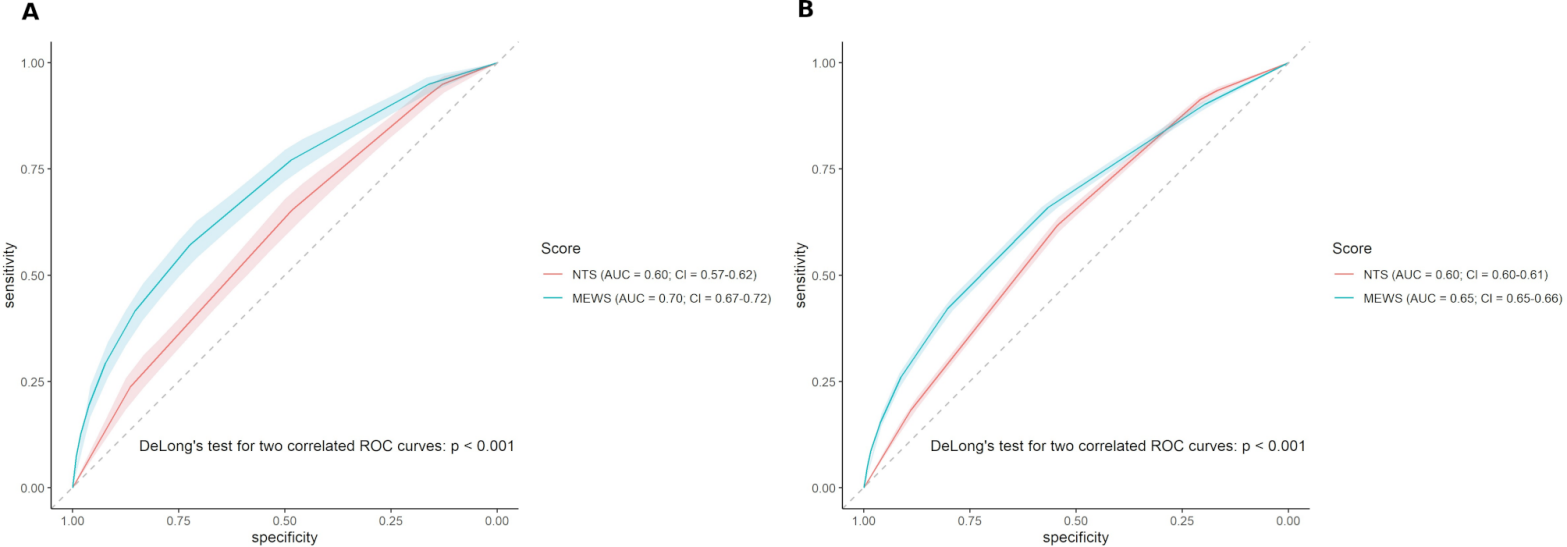
The receiver operator characteristics (ROC) curves and corresponding area under the curve (AUC) for both the Modified Early Warning Score (MEWS) and Netherlands Triage System (NTS) regarding 30-day mortality (A) or hospital admission (B).

The ROC curves are presented in figure 3. Figure 3A shows the MEWS score has a higher AUC for predicting 30-day all-cause mortality (0.70; 95% CI=0.67 to 0.72), compared with the NTS score (0.60; 95% CI=0.57 to 0.62) (p<0.001). In figure 3B, we see that the MEWS score also has a higher AUC for hospital admission (0.65; 95% CI=0.65 to 0.66), compared with the NTS score (0.60; 95% CI=0.60 to 0.61). (p<0.001).

## Discussion

We compared a traditional complaint-based triage scale and an EWS, represented by the NTS and MEWS score, respectively, on their ability to recognise patients in need of urgent care. The predictive performance of the MEWS score was significantly better than that of the NTS for 30-day mortality (0.70 vs 0.60; p<0.001) and hospital admission (0.65 vs 0.60; p<0.001), which are both well-studied surrogate markers for the urgent need of care. Furthermore, in instances with a particularly large discrepancy between the scores, the MEWS score seems to more accurately capture the urgency level that is warranted. Notably, neither tool reaches an excellent performance. While the MEWS reaches a fair (0.7–0.8) performance for 30-day mortality, all other AUCs can be considered poor (0.6–0.7).^9^

Complaint-based emergency triage scales such as the ESI, MTS and CTAS have been validated in at least 14 studies.^26^ A major challenge with the validation of these triage systems is the determination of an appropriate reference standard. The lack of a consensus definition about which patients actually require urgent care makes research in this field inherently difficult and limited in the ability to draw firm conclusions. In general, criterion validity and construct validity are the two main methodologies used to validate triage systems.

With criterion validity methods, performance of a triage system is compared with a reference standard, which is usually an expert panel.^26 30^ These studies report the validity of the triage scale as a function of the inter-rater agreement between the triagists and the expert panel and generally show fair agreement.^26 30^ Specifically for the NTS score, a recent study showed good agreement between triagists and an expert panel for 41 written cases.^31^

Although criterion validity methods could potentially detect true urgency best, they are labour intensive and cannot capture the full spectrum of clinical scenarios as seen in the ED.^26^ Given these limitations, and the fact that there is still significant subjectivity involved, researchers have usually opted for a method based on construct validity to validate triage tools, as we also did.^26^

With construct validity, surrogate markers that are deemed fair proxies for high urgency are used as outcome measures.^26^ These surrogates include but are not limited to admission rates, resource use, ED length of stay, overall costs and mortality rates. In the absence of a gold standard, construct validity methods have been named the ‘silver standard’ when it comes to validating triage systems.^30^ Studies generally show that the complaint-based triage scales like ESI, MTS and CTAS are associated with the surrogate markers for urgency. The most studied marker is hospital admission.^26^ The original validation study for the NTS score also showed significant associations of the NTS scores with hospital admission and resource use.^4^ MEWS scores and other EWS tools based on vital signs are actually created to detect the outcomes used as surrogate outcomes for urgent care needs. It is therefore no surprise that these models have good to excellent accuracy for detecting these outcomes.^9–14^

Our study adds to literature suggesting that EWS tools may have added clinical value in ED triage, either by augmenting or by replacing the current complaint-based triage scales. Several studies have explored the stand-alone use of EWS in ED triage, with the same surrogate endpoints we used.^9 18 20–22^ For example, Spencer and colleagues found AUCs of EWS scores for hospital admission ranging from 0.54 to 0.70 and Lee *et al* found AUCs of the MEWS for 30-day mortality of 0.779.^18 20^McCabe and colleagues specifically studied the use of an EWS in conjunction with the MTS.^19^ The study showed the EWS addition led to a more risk-adverse triage, but increased the overall ED length of stay, suggesting that these tools may work better separately.

The current study performed a direct comparison between a complaint-based triage scale and EWS, represented by the MEWS and NTS scores. Generally, these scores have much overlap and high NTS scores (non-urgent) never co-occur with high (urgent) MEWS. However, more urgent NTS scores do occur in combination with non-urgent MEWS scores. In these situations, the MEWS score seems to be more reflective of the urgency since the admission and mortality rates are lower than average in this group. Furthermore, the AUCs of the MEWS were significantly higher than those of the NTS for surrogate markers of urgent care needs.

Besides the performance of these scores, we believe the MEWS score is less complex and easier to use during triage since it consists of just eight items. Furthermore, from the distribution of the MEWS and NTS scores it appears that MEWS is better able to separate patients with lower from higher urgency. In our study, most needed, nearly half of the patients had an NTS score of 1 or 2, indicating the highest urgency. On the other hand, the right-skewed distribution of MEWS score found that most urgent cases are relatively rare and could be distinguished from lower urgency cases. Finally, using the MEWS score during triage will facilitate a continuous and comparable assessment over the course of hospital stay since it is also used in the hospital.

One aspect that favours the complaint-based approaches such as NTS is that they can be used to recognise specific conditions, such as acute angle-closure glaucoma or compartment syndrome, in which a short time-to-treatment is especially beneficial. In these situations, the urgency is not always reflected in a higher MEWS score as vital signs can be normal. However, currently used complaint-based triage systems have rarely been developed or validated in ways to show that these scores actually perform this function.

### Strengths and limitations

Our study has several strengths that distinguish this work from what has been published before. Through the use of deidentified EHR data, we were able to study a large population of patients which reflects a wide variety of clinical scenarios. The recorded MEWS and NTS scores were measured in the same patients at the same time, which lowers the chance that these results were biased. Other studies have often calculated clinical scores based on separate measurements, while our analysis is based on a structured data field that included a fully recorded MEWS score at the moment of triage.

Several limitations of the current study need to be addressed. As noted above, studies on triage urgency, including this one, are inherently limited by that fact that there is no gold standard for acuity. Our study used surrogate outcomes for urgent need of care, which are more reflective of severity of disease than urgent care needs. Since EWS tools are specifically created to detect poor outcomes, they may do better when we associate them with these surrogate markers rather than with ‘true’ urgency as assessed by an expert panel through criterion validity methods. Nevertheless, the criterion validity approach also has its limitations and subjectivity, as addressed in previous paragraphs.

Another limitation of our study is that it is a retrospective study with potential for selection bias. We only examined situations when both MEWS and NTS score were available, which may have resulted in more urgent patients being included. While we had documented NTS scores for 53 106 patients, we only had MEWS scores for 12 452 of those patients. However, we show that the distribution of NTS scores is similar in the complete population compared with the study population of patients who have both scores, indicating that missing MEWS scores occur across the spectrum of disease severity according to NTS. Furthermore, the overall distribution of MEWS scores in our population resembles the distribution in other cohorts.^14 20^

## Conclusion

We conclude that EWSs outperform currently used ED triage scales based on patient complaints regarding hospitalisation and 30-day mortality. In cases where these approaches yield particularly different urgency scores, the EWS, represented by the MEWS in our study, seems to assess the need for urgent care better than the complaint based NTS score. The results of this study suggest that EWSs could potentially replace the current emergency triage systems.

## Data Availability

Data are available on reasonable request.
