# Supplementary material for: Comparing complaint-based triage scales and early warning scores for emergency department triage
Source: Emerg Med J. 2022 Apr 13;39(9):691–6. doi: 10.1136/emermed-2021-211544 (PMC9411919; doi:10.1136/emermed-2021-211544)

Appendix

e-Tables

e-Table 1. Netherlands Triage System urgency scores, clinical meaning, and desired response.

NTS score	Condition	Response
**U0**	failure ABCD - resuscitation	Resuscitation (CPR)
**U1**	unstable ABCD – immediate danger to life	Immediate
**U2**	threatened ABCD or organ damage	As soon as possible
**U3**	real chance of harm / humane reasons	Within a few hours
**U4**	negligible risk of damage	Within 24 hours
**U5**	no chance of damage	Next workday

Schinkel M, *et al*. *Emerg Med J* 2022; 39:691–696. doi: 10.1136/emermed-2021-211544

e-Table 2. Modified Early Warning Scores as used in the VU university medical center .

**Score**	**3**	**2**	**1**	**0**	**1**	**2**	**3**
**Respiration rate**		< 9		9-14	15–20	21–30	>40
**Peripheral oxygen saturation (with therapy)**	< 90						
**Heart rate**		< 40	40–50	51–100	101–110	111–130	>130
**Systolic blood pressure**	< 70	70–80	81–100	101-200			
**Temperature**		< 35.1	35.1–36.5	36.5– 37.5	>37.5		
**Consciousness**				A	V	P	U
**Urine production**	< 75 mL in the last 4 hours: 1 point						
**Nurse being worried**	1 point						
A = Alert	V = Response to verbal stimulation			P = Response to painful stimulation		U = Unresponsive	

e-Figures

e-Figure 1. A bar chart of the absolute counts of the various Netherlands Triage System (NTS) scores complete population, before exclusion of patients without documented Modified Early Warning Scores (MEWS).

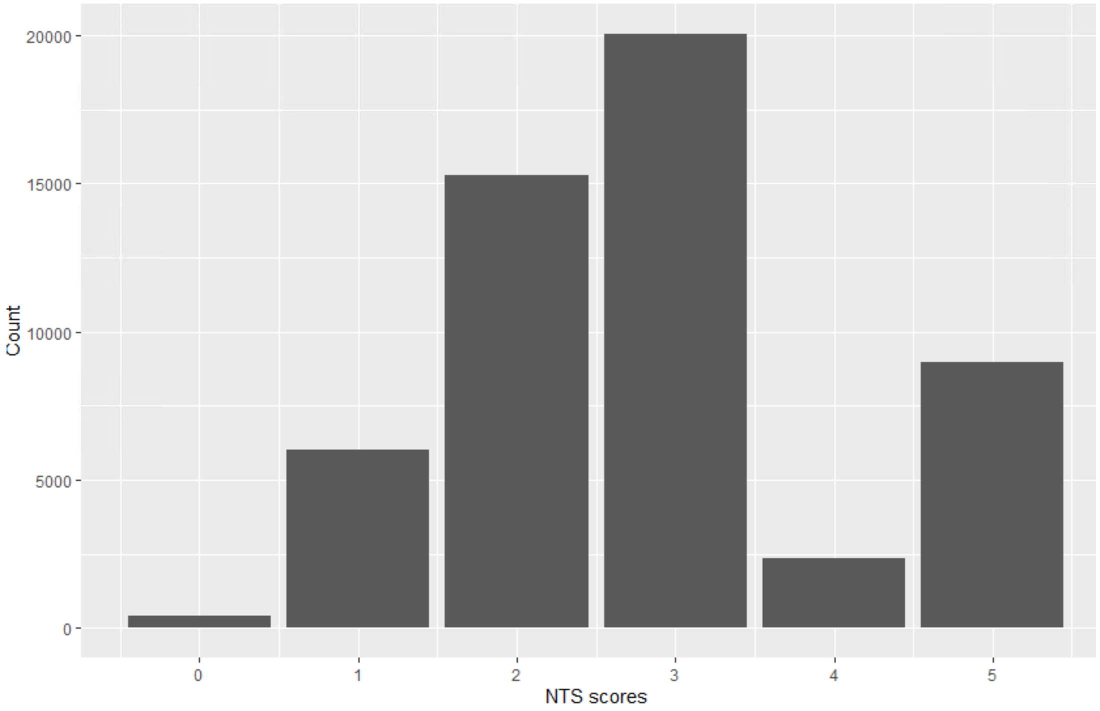

Supplement: Supplementary data [file emermed-2021-211544supp001.pdf]
